# The immunogenicity and protective immunity of multi-epitopes DNA prime-protein  boost vaccines encoding Amastin-Kmp-11, Kmp11-Gp63 and Amastin-Gp63 against visceral leishmaniasis

**DOI:** 10.1371/journal.pone.0230381

**Published:** 2020-03-16

**Authors:** Jianhui Zhang, Jinlei He, Jiao Li, Qi Zhou, Han Chen, Zhiwan Zheng, Qiwei Chen, Dali Chen, Jianping Chen

**Affiliations:** 1 Department of Pathogenic Biology, West China School of Basic Medical Sciences and Forensic Medicine, Sichuan University, Chengdu, Sichuan, China; 2 Animal Disease Prevention and Food Safety Key Laboratory of Sichuan Province, Sichuan University, Chengdu, Sichuan, China; Instituto Butantan, BRAZIL

## Abstract

Visceral leishmaniasis (VL) is the most fatal form of leishmaniasis if left untreated and 50,000 to 90,000 new cases of VL occur worldwide each year. Although various vaccines had been studied in animal models, none of them was eligible to prevent human from infections. In this study, according to the silico analysis of *Leishmania* Amastin, Kmp-11 and Gp63 protein, dominant epitope sequences of these proteins were selected and linked to construct dominant multi-epitopes DNA and protein vaccines (Amastin-Kmp-11, Amastin-Gp63 and Kmp-11-Gp63) against VL. BALB/c mice were immunized with a DNA prime-protein boost immunization strategy and challenged with a new *Leishmania* parasite strain isolated from a VL patient. After immunization, the results including specific antibody titers, IL-4 and TNF-α levels, and CD4 and CD8 T cell proportion suggested the potent immunogenicity of the three vaccines. After infection, the results of spleen parasite burdens in the three vaccine groups were significantly lower than those of control groups, and the parasite reduction rates of Amastin-Kmp-11, Amastin-Gp63 and Kmp-11-Gp63 groups were 89.38%, 91.01% and 88.42%, respectively. Spleen smear observation and liver histopathological changes showed that all vaccine groups could produce significant immunoprotection against VL and Amastin-Gp63 vaccine was the best. In conclusion, our work demonstrated that the three dominant multi-epitopes Amastin-Kmp-11, Amastin-Gp63 and Kmp-11-Gp63 DNA prime-protein boost vaccines might be new vaccine candidates for VL, and the Amastin-Gp63 vaccine have best efficacy.

## Introduction

Leishmaniasis is caused by *Leishmania* protozoan with sand flies as the medium of transmission. *Leishmania* protozoan is present in the form of promastigotes in sand fly, invading humans or vertebrates through the bite of sand fly, and parasitizing host macrophages in the form of amastigotes. There are three forms of leishmaniasis: visceral leishmaniasis (VL), mucocutaneous leishmaniasis, and cutaneous leishmaniasis, of which visceral leishmaniasis is the most fatal if left untreated. Visceral leishmaniasis, caused by *Leishmania donovani (L*. *donovani)*, *Leishmania infantum (L*. *infantum*, and also known as *Leishmania chagasi*, *L*. *chagasi)*, is characterized with irregular bouts of fever, weight loss, Hepatosplenomegaly and anemia. According to the World Health Organization, 50,000 to 90,000 new cases of VL occur worldwide each year, and more than 95% of new cases in 2017 were distributed in Bangladesh, Brazil, China, Ethiopia, India, Kenya, Nepal, Somalia, South Sudan and Sudan [1]. At present, the treatment of leishmaniasis primarily relies on chemical drugs, such as pentavalent antimonials and amphotericin B, but these treatments may bring parasite resistance, side effects and high costs [2–4]. The invasion of *Leishmania* can produce a relatively long-term immune memory in the host, which prevents the cured patients from reinfection with *Leishmania* [5, 6]. Therefore, it is a wise choice to develop prophylactic and therapeutic vaccines against VL. However, at present, there is no effective vaccine against VL that can be used in humans, and researchers still need to explore and develop an effective vaccine [2, 7].

The progression of leishmaniasis depends not only on parasite strain but also on the host’s immune responses including Th1 and Th2 responses [2]. *Leishmania* parasites can invade macrophages and escape immune attacks by inhibiting the activation of macrophages [8]. After *Leishmania* infection, the CD4^+^ T helper type 1 cells (Th1 cells) of polarized Th1 immune responses secrete Th1 type cytokines (IL-12, INF-γ and TNF-α), activate CD8^+^ T cells and macrophages, promote the formation of hepatic granulomas, upregulate nitric oxide and stimulate the activation of oxidative burst to kill intracellular *Leishmania* parasites [9, 10]. However, IL-4 produced by CD4^+^ T helper type 2 cells (Th2 cells) in Th2 responses inhibits Th1 responses and macrophage activation, which helps parasites survive and results in susceptibility of the host to severe infection. Some studies have shown that the role of Th2 immune responses in resisting *Leishmania* in host cannot be completely denied, due to the ability of IL-4 to promote the secretion of IL-12 and INF-γ in the early stage [11–13]. Th2 cells also produce another essential suppressive cytokine IL-10 that inhibits DC migration to T cell areas, suppresses Th1 responses, macrophage activation and control excessive detrimental inflammatory. IL-10 is responsible for immunological dysfunction and architectural damage in spleen, which inhibits proinflammatory responses [9, 14, 15]. In belief, the outcomes of VL development is determined by the balance between Th1 and Th2 responses [2, 16, 17].

Gp63, a glycoprotein of 63 KDa, is a conserved protein and is highly expressed on the surface of promastigotes of many *Leishmania* parasite species. Gp63 is also a virulence factor helping *Leishmania* translocate into macrophages, and thought to be the significant antigen recognized by antibodies in the serum of patients [18, 19]. Studies have shown that Gp63 as a DNA vaccine or recombinant protein vaccine combined with an adjuvant can cause a strong Th1 response and excellent immune protection [20–22]. Kinetoplastid membrane protein (Kmp-11), expressed in both amastigotes and promastigotes, is also a highly conserved protein closely related to membrane structure in all members of the kinetoplastid, which also showed high immunogenicity in mice, rats and dogs [23]. Kmp-11, as a DNA vaccination, showed mixed Th1 and Th2 responses and inducing complete protection against VL in a hamster model [24]. In addition, mice were immunized with Kmp-11 12–31aa peptide +CpG ODNs-pulsed bone marrow-derived dendritic cells to explore the resistance to VL, and results showed the mice had higher immune protection accompanied by high levels of INF-γ and IL-17 [23]. Amastin, a group of surface glycoproteins expressed in *Leishmania* amastigotes, contains 11aa at positions 52–62 that are highly conserved and unique to trypanosomatid protozoa. De Paiva et al speculated that amastin might participate in the tight contact between the surface of wild type amastigotes and the membrane of the macrophage parasitophorous vacuole [25]. Stober et al showed amastin proteins at positions 1–63 containing the amastin signature sequence is highly immunogenic and possess good protection in mice [26]. Sima et al found that amastin protein could be used as a relevant immune biomarker for serodiagnosis of VL cases at different stages of the disease [27].

The third-generation vaccine, DNA vaccine, can induce better cellular and humoral immunity compared to protein vaccine, can trigger a strong Th1 response, and maintains long-term immune memory. DNA vaccine fusion genes can create multiple antigens and single multifunctional vaccines [28]. Traditional homologous prime-boost immunization is vaccinated multiple times by the same type of vaccines. Heterologous prime-boost immunization is carried out with different types of vaccines containing the same antigen [29]. Many successful vaccines were given by traditional homologous prime-boost immunization, such as Diphtheria, Tetanus and Pertussis (DTP) vaccine [29]. However, some studies showed that heterologous prime-boost vaccines are more immunogenic than homologous prime-boost vaccines and the efficacy of DNA prime -protein boost is the best [29–31].

HLA-A2 and HLA-A24 molecules, belong to the human major histocompatibility complex class Ⅰ (MHC Ⅰ), which present antigens to CD8^+^ T cells and play important roles in human health maintenance. HLA-A2 expresses at high frequency in various ethnic groups, including Asians and Caucasians [32]. HLA-A24 is also widely presented in human populations, especially Asian populations [33]. HLA-DR are the most expressed molecules in the human major histocompatibility complex class Ⅱ (MHC Ⅱ), which bind microbial peptides in an endosomal compartment and present them on the surface of professional antigen presenting cells (APCs) for CD4 T cell surveillance [34, 35]. HLA-DR1 molecule can bind the peptides that are also recognized by other DR molecules, including DR4 and DR7 [36]. Correspondingly, HLA-A2, HLA-A24 and HLA-DR1 have been used for designing peptide-based vaccines [37–39]. In addition, in order to ensure the effectiveness of mouse experiments, we also need to consider that vaccine antigens should be recognized by MHC molecules of mice. It has been reported that H2-Db, a mouse MHC class Ⅰmolecule, can enhance the stimulation of mouse CD8^+^ T cells by presenting protein epitopes [40]. Therefore, based on the fragments rich in HLA-A2, HLA-A24, HLA-DR1 and H2-Db molecules of antigens, combined with the results of bioinformatics analysis, we comprehensively screened the dominant epitope fragments for vaccine preparation.

In this study, we used Gp63, Kmp-11 and Amastin protein as pre-selected antigens to explore effective multi-epitopes DNA prime-protein boost vaccines against VL. According to the silico analysis of Amastin, Kmp-11 and Gp63 from *Leishmania*, we selected the amino acid sequences of Amastin 1-71aa, Kmp-11 1-91aa and Gp63 138-360aa and linked them to each other to construct dominant multi-epitopes DNA and protein vaccines (Amastin-Kmp-11, Amastin-Gp63 and Kmp-11-Gp63). To assess the immunogenicity and immunoprotection of these vaccines, BALB/c mice, susceptible to infection and suitable for vaccine animal experiments, were vaccinated with DNA prime-protein boost immunization strategy and challenged with *L*. *infantum*. Our study combined epitopes vaccine and DNA prime-protein boost vaccine to develop vaccines against VL, which provides a basis for the study of leishmaniasis and other infectious disease vaccines.

## Materials and methods

### Animals and parasites

All experimental operations of animals in our study were performed following the ethical rules and approved by Sichuan University Medical Ethics Committee (Approval Number: K2018056). Two golden hamsters (Mesocricetus auratus, 12-week-old, female, Chengdu Institute of Biological Products Co., Ltd, China.) and 156 BALB/c mice (8-week-old, female, Dassy experimental animals Co., Ltd, Chengdu, China.) were maintained under standard conditions (temperature: 20~26°C, humidity: 40–70%, 12h light and 12h dark, soft floor mat, every 12 BALB/c mice were housed in a 46×30×16cm shelter and two golden hamsters were housed in a 32×21×16cm shelter). Animals had free access to water and were fed a standard animal diet once a day. All mice were evaluated their well-being by free movement, good appetite, no hyperemia of conjunctiva, no secretion of pupil and external genitalia, no bulge in abdomen, clean and glossy soft fur, and normal weight and temperature. Mice were monitored daily to assess health during the immunization and parasite challenge. After experiment, the animals were euthanized by intraperitoneal injection of pentobarbital sodium and cervical dislocation, and none of them need post-operative care.

The *L*. *infantum* (MHOM/CN/2016/SCHCZ) used in this study was isolated from the bone marrow of a clinical patient and kept in golden hamsters in our laboratory. Golden hamsters were euthanized by intraperitoneal injection of pentobarbital sodium and cervical dislocation, and the *L*. *infantum* was isolated from the spleen of golden hamsters infected for six months. The spleen homogenate was added into M199 (HyClone, USA) medium with 10% Fetal Bovine Serum (Gibco, USA) to incubate until the density of promastigotes was 1ⅹ10^7^/ml. The stationary phase promastigotes were obtained by centrifugation and resuspended with phosphate buffer saline (PBS). The promastigotes were collected to freeze and thaw for three times to prepare soluble antigen (SLA). Promastigotes were also used to extracted DNA by TIANamp Genomic DNA Kit (TIANGEN, China) according to manufacturer’s instructions.

### Bioinformatics analysis

As described in previous studies [41, 42], amino acid sequences of Amastin (GenBank ID: MH107836) and Gp63 (GenBank ID: Z83677.1) of *L*. *infantum* were employed for bioinformatics analysis. Secondary structures and surface properties of Amastin and Gp63 were analyzed by Protean module of DNASTAR software to predict antigenic index, and the amino acid fragments of the proteins with high antigenic index were selected. The HLA-A2, HLA-A24, HLA-DR1 and H2-Db epitopes of selected amino acid fragments were predicted using online analysis systems (SYFPEITHI: http://www.syfpeithi.de/bin/mhcserver.dll/epitopeprediction.htm; Rankpep: http://imed.med.ucm.es/Tools/rankpep.htmL; NetCTL 1.2 server: http://www.cbs.dtu.dk/services/NetCTL/; NetMHC 4.0 server: http://www.cbs.dtu.dk/services/NetMHC/). The tertiary structures and binding sites of selected amino acid fragments were predicted using PHYRE2 (http://www.sbg.bio.ic.ac.uk/phyre2/html/page.cgi?id=index) and 3DLigandSite (http://www.sbg.bio.ic.ac.uk/~3dligandsite/), respectively. As for Kmp-11 protein (GenBank ID: MH107835), the analytic results were described in our early study [41]. Combining the above bioinformatics analysis results, Amastin 1–71 aa, Kmp-11 1–92 aa and Gp63 138–360 aa were selected as antigens to prepare dominant multi-epitopes DNA and protein vaccines.

### DNA preparation

With the primers listed in Table 1, the antigenic gene fragments selected from *Amastin* (a), *Kmp-11* (k) and *Gp63* (g) was amplified and linked by overlapping polymerase chain reaction (PCR), using the genomic DNA of *L*. *infantum* as the template. The overlapping PCR was performed by three steps. The first step was to obtain prior genes (a-linker, linker-k, linker-g and k-linker), and the PCR productions were electrophoresed in 1.5% agarose gel and purified using Universal DNA Purification Kit (TIANGEN, China). The second step was designed to link prior genes to obtain a-k, a-g, k-g linked genes. The third step was to amplify the linked genes, and the PCR productions were purified. After obtaining the purified linked genes, they were cloned into pET32a(+) plasmids (Biodee, China) to construct prokaryotic recombinant plasmids (pET32a-a-k, pET32a-a-g and pET32a-k-g). To construct eukaryotic recombinant plasmids, the pET32a-a-k, pET32a-a-g and pET32a-k-g plasmids were used as templates to amplify new a-k, a-g and k-g linked genes with primers containing Kozak sequences and stop codon (af and kr for a-k, af and gr for a-g, and kf and gr for k-g). The eukaryotic plasmid pVAX1 (Wuhan Miaoling Bioscience & Technology Co., China) was used to construct eukaryotic recombinant plasmids (pVAX1-a-k, pVAX1-a-g, pVAX1-k-g). Subsequently, the eukaryotic and prokaryotic recombinant plasmids were transfected into competent DH5α cells for identification by colony PCR, DNA sequencing and BLAST. The positive recombinant plasmids were extracted using EndoFree Maxi plasmid Kit (TIANGEN, China).

**Table 1:** Primers used in PCR.

Plasmids	Gene name	Primer sequences: 5´–3´		Size	Restriction enzymes
	linker	GGT GGC GGT GGA AGC GGC GGT GGC GGA AGC GGC GGT GGC GGC AGC		45bp	
pET32a(+)	a-linker	AF:	CGC **GGA TCC** ATG CTG TGC TCG TGC ATC	258bp	*Bam*HⅠ
		ARL:	GCT GCC GCC ACC GCC GCT TCC GCC ACC GCC GCT TCC ACC GCC ACC CA CCT GGA ACA GCT GCT		
pET32a(+)	k-linker	KF:	CGC **GGA TCC** ATG GCC ACC ACG TAC GAG GA	322bp	*Bam*HⅠ
		KRL:	GCT GCC GCC ACC GCC GCT TCC GCC ACC GCC GCT TCC ACC GCC ACC CTT GGA CGG GTA CTG CGC		
pET32a(+)	linker-k	KFL:	GGT GGC GGT GGA AGC GGC GGT GGC GGA AGC GGC GGT GGC GGC AGCATG GCC ACC ACG TAC GAG GAC	322bp	
		KR:	CCC **AAG CTT** CTT GGA CGG GTA CTG CG		*Hind*Ⅲ
pET32a(+)	linker-g	GFL:	GGT GGC GGT GGA AGC GGC GGT GGC GGA AGC GGC GGT GGC GGC AGC GAG AAG CGC GAC ATC CTG	714bp	
		GR:	CCC **AAG CTT** GCT GAA GTC CGC CTG GTA G		*Hind*Ⅲ
pVAX1	a-k	af:	CCC **AAG CTT**GCCACCATGGGG ATG CTG TGC TCG TGC ATC	585bp	*Hind*Ⅲ
		kr:	CGC **GGA TCC** TTA GTG GTG GTG GTG GTG GTG CTT GGA CGG GTA CTG CGC		*Bam*HⅠ
pVAX1	a-g	kf:	CCC **AAG CTT**GCCACCATGGGG ATG GCC ACC ACG TAC GAG GA	978bp	*Hind*Ⅲ
		gr:	CGC **GGA TCC** TTA GTG GTG GTG GTG GTG GTG GCT GAA GTC CGC CTG GTA G		*Bam*HⅠ
pVAX1	k-g	kf:	CCC **AAG CTT**GCCACCATGGGG ATG GCC ACC ACG TAC GAG GA	1041bp	*Hind*Ⅲ
		gr:	CGC **GGA TCC** TTA GTG GTG GTG GTG GTG GTG GCT GAA GTC CGC CTG GTA G		*Bam*HⅠ

The restriction enzyme sites are in bold. The Kozak sequences are underlined.

### Prokaryotic expression of recombinant proteins in *E*. *coil*

Prokaryotic recombinant plasmids (pET32a-a-k, pET32a-a-g and pET32a-k-g) were transfected into competent *E*. *coil* BL21 (TIANGEN, China), and induced by 1mM isopropyl-β-D- thiogalactopyranoside (IPTG, Solarbio, USA) for 6h culture to express His-tag fusion multi-epitopes protein (pAmastin-Kmp-11, pAmastin-Gp63 and pKmp-11-Gp63). Then, *E*. *coil* BL21 were collected for three repeated freeze-thaw and ultrasonic disruption, and the supernatant was used to determine the expression of the proteins. The inclusion bodies from cells lysate were solubilized by adding urea and dialyzed with dialysis membrane in refolding buffer (50mM Tris, 50mM NaCl, 0.5mM EDTA, 5% glycerinum) and PBS. The soluble proteins were purified using HisTrap affinity columns (GE, Healthcare, USA). The purified proteins were detected by sodium dodecyl sulfate-polyacrylamide gel electrophoresis (SDS-PAGE) and Western blot. His-tag mouse monoclonal antibody (SAB, USA, 1:5000) was used as the primary antibody, and the secondary antibody was horseradish-peroxidase conjugated goat anti-mouse lgG monoclonal antibody (ZAGB-BIO, China, 1:4000). Eventually, the Affinity® ECL kit was employed to reveal the blotted proteins. Endotoxin of purified proteins was tested using Endotoxin Detection Limulus Kit (Bioendo, Xiamen, China) and the eligible purified proteins were prepared for protein vaccine and ELISA.

### Eukaryotic expression of recombinant proteins in NIH3T3 cells and BALB/c mice hind leg quadriceps

NIH3T3 cells (GuangZhou Jennio Biotech Co., China) were cultured in DMEM-H medium with 10% Fetal Bovine Serum, 100IU/ml penicillin and 100mg/ml streptomycin at 37°C and 5% CO_2_. Eukaryotic recombinant plasmids (pVAX1-a-k, pVAX1-a-g, pVAX1-k-g) were mixed with lipofectamine 2000 (Invitrogen, USA) and transfected into NIH3T3 cells when the cells on 24-well plates were 80% confluent. After 48 hours of culture, cells were washed thrice with PBS and then lysed by Cell lysis buffer (Beyotime, China) to confirm the expression of eukaryotic recombinant plasmids using western blot. The primary antibody and secondary antibody used for western blot were consistent with those used in prokaryotic expression experiments. Reverse transcription polymerase chain reaction (RT-PCR) was also used to identify the expression of eukaryotic recombinant plasmids. The total RNA of NIH3T3 cells was extracted using Cell Total RNA Isolation Kit (Foregene, China) and employed to synthesize the first strand cDNA using RevertAid First Strand cDNA Synthesis Kit (ThermoFisher) with random primers. Subsequently, the cDNA was employed for PCR amplification (primers: af and kr for pVAX1-a-k, af and gr for pVAX1-a-g, and kf and gr for pVAX1-k-g) (Table 1), and the PCR productions were detected by agarose gel electrophoresis.

Two weeks after primary immunization (please refer to ‘Immunization and parasites challenge of mice’ section), three BALB/c mice of G2, G3, G4 and G5 were sacrificed to collect intramuscular tissue in right hind leg quadriceps for RT-PCR and immunofluorescence. As for immunofluorescence, the His-tag sequences in eukaryotic recombinant plasmids were replaced with myc-tag. Myc-tag mouse monoclonal antibody (Cell Signaling, USA, 1:500) was used as the primary antibody, and the secondary antibody was a fluorescein-conjugated affinipure goat anti-mouse IgG (Servicebio, Wuhan, China,1 : 400).

### Immunization and parasites challenge of mice

BALB/c mice were divided into one normal group (G0) without any operations, two control groups (G1: PBS/Freund's complete adjuvant (FCA) and G2: PBS) and three vaccine groups (G3: Amastin-Kmp-11, G4: Amastin-Gp63 and G5: Kmp-11-Gp63) (n = 27 per group). The mice of G1, G2, G3, G4 and G5 groups were intramuscularly injected with 50μl 0.5% Lidocaine in both hind leg quadriceps. After three days, G1 and G2 groups were intramuscularly injected with sterile PBS, and G3, G4 and G5 groups were respectively intramuscularly injected with 50μg DNA (pVAX1-a-k, pVAX1a-g and pVAX1-k-g) in both hind leg quadriceps for primary immunization. After three weeks, G1 and G2 groups respectively received sterile FCA and PBS. For boost immunization, G3, G4 and G5 groups were subcutaneously injected with 15μg purified multi-epitopes protein (pAmastin-Kmp-11, pAmastin-Gp63 and pKmp-11-Gp63) mixed with FCA, respectively. At 3rd, 6th and 9th week post-immunization, four mice per group were sacrificed by intraperitoneal injections of pentobarbital sodium and cervical dislocation to collect serum and spleen for analysis. At 3rd week after boost immunization, the remaining 12 mice of each group, except group G0, were challenged with promastigotes of *L*. *infantum* (1ⅹ10^6^ per mouse) through intraperitoneal injections. Four mice per group were euthanized at 4th, 8th and 12th week post-infection to collect serum, spleen and liver for analysis, respectively.

### ELISA of serum lgG, lgG1, lgG2a and cytokines

Serum samples from each group, gained at 3rd, 6th and 9th week post-immunization and at 4th, 8th and 12th week post-infection, were evaluated by enzyme linked immunosorbent assay (ELISA) to detect specific antibody titers. The protocol of ELISA was referenced from previous study [42]. We performed 2μg/ml, 5μg/ml, 10μg/ml and 20μg/ml concentration of antigen protein (pAmastin-Kmp-11, pAmastin-Gp63, pKmp-11-Gp63, and SLA) for coating, and determined that 2μg/ml was the best coating concentration using checkerboard titration. Serum samples were serially diluted twofold from 1:16000 to 1:8192000. Horseradish peroxidase-conjugated goat anti-mouse lgG (absin, China, 1:10000), lgG1 (Proteintech, USA, 1:1000) and lgG2a monoclonal antibodies (Proteintech, USA, 1:1000) were used as secondary antibodies. Single component 3,3',5,5'-Tetramethylbenzidine reagent was used as a substrate for color rendering. The reaction was stopped by adding 1 M HCl, and the absorbance was measured at 450 nm. Cytokines IL-4 and TNF-α in serum of each group were evaluated using OptEIA^™^ Set IL-4 or TNF (BD, USA) at 4th, 8th and 12th week post-infection.

### Analysis of CD4^+^ and CD8^+^ lymphocyte subsets using flow cytometry

To analyze the classification of lymphocyte subsets (CD3^+^CD4^+^ cells, CD3^+^CD8^+^ cells and CD3^+^CD4^-^CD8^-^ cells), splenocytes from each group at 3rd week post-immunization and 8th week post-infection were prepared in 100μl (10^6^ cells/ml) PBS and incubated with hamster anti-mouse CD3 PE/CY7, rabbit anti-mouse CD4 APC and rabbit anti-mouse CD8 FITC antibodies (BD, USA) for 30 min at 4°C. Splenocytes were washed twice with PBS and resuspended in 400μl PBS. Finally, splenocytes were detected using CytoFLEX flow cytometry and analyzed by CytExperit software.

### Evaluation of parasite burdens

Quantitative real-time PCR (qPCR) was carried out to determine the parasite loads according to previous study [43]. The target gene, *Leishmania* cysteine protease B (CPB), is a highly expressed and stable gene with five copies in *Leishmania* [44]. It was amplified by PCR using the total DNA of *L*. *infantum* as a template (Primers: F: 5' AACGAAACGGTTATGGCTGC 3', R: 5' CTTGTTGTACCCGACGAGCA 3'). The PCR products (CPB DNA) were purified and measured by NanoDrop 2000C (Thermofisher, USA), and were serially diluted from 1:10 to 1:10,000,000 for the preparation of qPCR standard curve. To estimate the parasite burdens, the total DNA of spleens in each group was extracted as the template to amplify CPB gene by qPCR at 8th and 12th week post-infection. The qPCR results were analyzed by Bio-Rad CFX Manager software to quantify CPB gene copies from 100ng total DNA by converting cT value into standard curve. Finally, the parasite burdens were determined by the number of CPB copies per 100ng of total spleen DNA in each group [45]. Moreover, spleens from each group at 12th week post-infection were made into smears and stained by Wright’s staining.

### Pathological changes of liver

Livers harvested from each group at 4th, 8th and 12th post-infection were fixed by formalin, paraffined, made into sections and stained with haematoxylin-eosin. The pathological changes of liver in each group were observed using light microscopy.

### Statistical analysis

Statistical analyses were performed using IBM SPSS Statistics 22 version. One-way analysis of variance was used to analyze data of animal experiments. The statistical difference was designed as asterisks (*P<0.05, **P<0.01, ***P<0.001).

## Results

### Bioinformatics analysis of each protein

**Fig 1:**
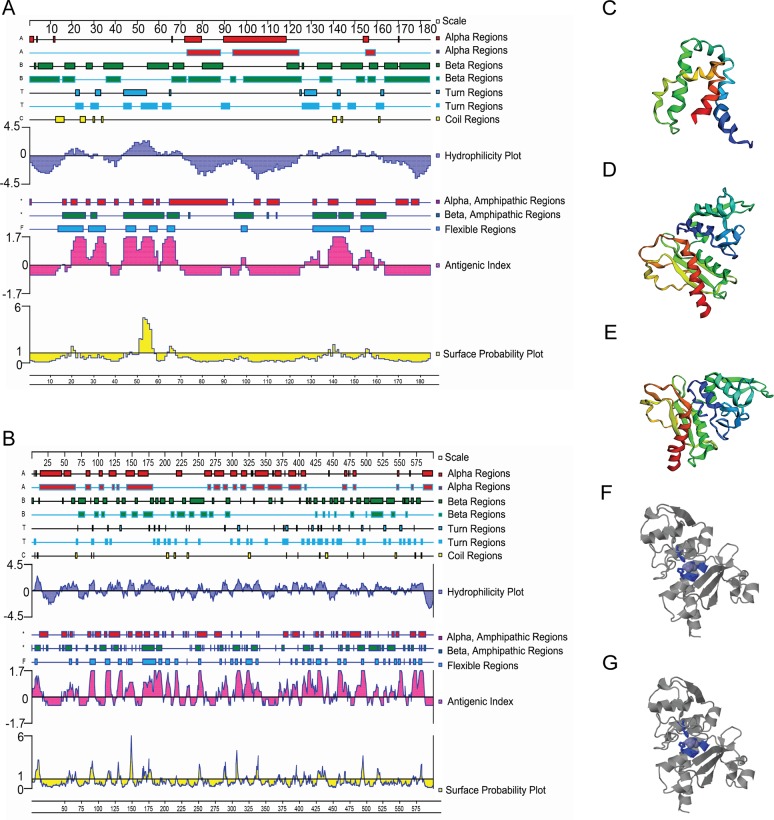
Secondary and tertiary structures and potential binding sites of the recombinant proteins. (A) Amastin Secondary structure containing surface properties. (B) Gp63 Secondary structure containing surface properties. (C) Duplex protein Amastin-Kmp-11 tertiary structures. Image is coloured by rainbow N → C terminus. Model dimensions Model dimensions (Å): X:56.047 Y:34.951 Z:66.482. A total of 85% of the residues were modelled with 98.4% confidence. (D) Duplex protein Amastin-Gp63 tertiary structures. Image is coloured by rainbow N → C terminus. Model dimensions Model dimensions (Å): X:46.104 Y:49.312 Z:55.074. A total of 72% of the residues were modelled with 100% confidence. (E) Duplex protein Kmp-11-Gp63 tertiary structures. Image is coloured by rainbow N → C terminus. Model dimensions Model dimensions (Å): X:46.104 Y:49.312 Z:55.074. A total of 65% of the residues were modelled with 100% confidence. (F) Potential binding sites of duplex protein Amastin-Gp63. Four potential binding sites 200HIS, 201GLU, 204HIS and 270HIS were predicted and coloured by blue. (G) Potential binding sites of duplex protein Kmp-11-Gp63. Four potential binding sites 221HIS, 222GLU, 225HIS and 291HIS were predicted coloured by blue.

Secondary structures and surface properties of Amastin and Gp63 protein were analyzed, including α-helix, β-sheet, β-turn, coil region, hydrophilicity, flexibility, surface probability and antigenic index. The analytic results of Kmp-11 protein were described in our previous study [41]. Amastin 1–71 aa, Kmp-11 1–91 aa and Gp63 138–360 aa were mainly β-turn and coil region with high hydrophilicity, flexibility and surface probability, which led to high antigenic index (Fig 1A and 1B). These amino acid fragments were selected and linked for HLA and H2-Db epitopes prediction. The results suggested that Amastin-Gp63 protein had the largest number of HLA-A2, HLA-A24, HLA-DR1 and H2-Db epitopes (Tables 2, 3, 4 and 5). The tertiary structures of Amastin-Kmp-11, Amastin-Gp63 and Kmp-11-Gp63 protein are showed in Fig 1C, 1D and 1E, respectively. The potential binding sites of Amastin-Gp63 and Kmp-11-Gp63 are showed in Fig 1F and 1G, respectively.

**Table 2:** Prediction of HLA-A24, HLA-A2 and HLA-DR1 epitopes of Amastin-Gp63.

MHC supertypes	SYFPEITHI		Rankpep		NetCTL 1.2 server		NetMHC 4.0 server	
HLA-A24	2	LCSCIVFMFL			2	LCSCIVFMF	2	LCSCIVFMF
	94	KYLIPQALQL			9	MFLVTSAPI	9	MFLVTSAPI
	247	QYGCGTLEYL			52	NYDYRPTSI	303	FYQADFSKL
	303	FYQADFSKL			161	AWATTCQVF	161	AWATTCQVF
	289	YYSALTMAIF			189	RYDQLVTRV	189	RYDQLVTRV
	189	RYDQLVTRV			289	YYSALTMAI	289	YYSALTMAI
					290	YSALTMAIF	290	YSALTMAIF
					303	FYQADFSKL		
HLA-A2	95	YLIPQALQL			95	YLIPQALQL	95	YLIPQALQL
	217	RILESISNV			217	RILESISNV	217	RILESISNV
	100	ALQLHTERL			100	ALQLHTERL	276	AQDELMAPA
	293	LTMAIFQDL			293	LTMAIFQDL	298	FQDLGFYQA
	160	LAWATTCQV			160	LAWATTCQV	1	MLCSCIVFM
	1	MLCSCIVFM			1	MLCSCIVFM		
	202	MAHALGFSV			202	MAHALGFSV		
	185	NIASRYDQL						
	272	KMRNAQDE						
	198	VTHEMAHAL						
	31	GGASKLSCV						
	136	HITDGLSNT						
	145	DFVMYVASV						
	192	QLVTRVVTH						
HLA-DR1			729	KRQIYVAAF				
			19	RWLCAGALV				
			301	DAQKLLEKM				
			406	KKEGWRPRR				
			370	RYVILGGHR				
			433	EWAEENSRL				
			207	KVFRGNKVK				
			660	VLRMMNDQL				
			571	KYHLTVAQV				
			298	GYYDAQKLL				
			732	IYVAAFTVQ				
			343	KMHIHSTNE				
			212	NKVKNAQLA				
			215	NKVKNAQLA				
			199	KIVIARYGK				

The results contain residues numbers and peptide sequence, which have been analyzed online by using four different HLA epitopes prediction systems. The results that are simultaneously predicted by more than three systems are marked with underline.

**Table 3:** Prediction of HLA-A24, HLA-A2 and HLA-DR1 epitopes of Kmp-11-Gp63.

MHC supertypes	SYFPEITHI		Rankpep		NetCTL 1.2 server		NetMHC 4.0 server	
HLA-A24	4	TYEEFSAKL			4	TYEEFSAKL	4	TYEEFSAKL
	52	RMIKHTEKF			47	HYEKFERMI	52	RMIKHTEKF
	114	KYLIPQALQ			182	AWATTCQVF	182	AWATTCQVF
	72	HFKQKFAEL			210	RYDQLVTRV	210	RYDQLVTRV
	310	YYSALTMAI			310	YYSALTMAI	310	YYSALTMAI
	268	QYGCGTLEY			311	YSALTMAIF	268	QYGCGTLEY
	324	FYQADFSKL			324	FYQADFSKL	324	FYQADFSKL
							114	KYLIPQALQ
							221	THEMAHALG
HLA-A2	116	YLIPQALQL			116	YLIPQALQL	116	YLIPQALQL
	238	RILESISNV			238	RILESISNV	238	RILESISNV
	181	LAWATTCQV			181	LAWATTCQV	300	FQDLGFYQA
	223	MAHALGFSV			223	MAHALGFSV	297	AQDELMAPA
	121	ALQLHTERL			121	ALQLHTERL		
	314	LTMAIFQDL			314	LTMAIFQDL		
	293	KMRNAQDEL						
	206	NIASRYDQL						
	219	VTHEMAHAL						
	157	HITDGLSNT						
	166	DFVMYVASV						
	213	QLVTRVVTH						
	255	VINSSTAVA						
HLA-DR1			22	KMQEQNAKF				
			53	RMIKEHTEK				
			309	GYYSALTMA				
			210	RYDQLVTRV				
			220	THEMAHALG				
			149	GHFKVPPAH				
			73	FKQKFAELL				
			310	YYSALTMAI				
			84	QKAAQYPSK				
			61	KFNKKMHEH				
			179	DVLAWATTC				
			324	FYQADFSKL				
			127	ERLKVRQVQ				
			115	KYLIPQALQ				
			290	SHIKMRNAQ				
			130	KVRQVQDKW				
			160	DFVMYVASV				

The results contain residues numbers and peptide sequence, which have been analyzed online by using four different HLA epitopes prediction systems. The results that are simultaneously predicted by three systems are marked with underline.

**Table 4:** Prediction of HLA-A24, HLA-A2 and HLA-DR1 epitopes of Amastin- Kmp-11.

MHC supertypes	SYFPEITHI		Rankpep		NetCTL 1.2 server		NetMHC 4.0 server	
HLA-A24	158	HFKQKFAELL			2	LCSCIVFMF	2	LCSCIVFMF
	139	RMIKHTEKF			9	MFLVTSAPI	90	SMATTYEEF
	89	TTYEEFSAKL			52	NYDYRPTSI	139	RMIKHTEKF
	90	TYEEFSAKLD			133	HYEKFERMI	1	MLCSCIVFM
					90	SMATTYEEF		
HLA-A2	1	MLCSCIVFM			1	MLCSCIVFM	1	MLCSCIVFM
	89	TTYEEFSAKL						
	8	FMFLVTSAPI						
	32	GASKLSCVTV						
	59	SIGCARSKQL						
	62	CARSKQLFQV						
	171	KAAQYPSKKL						
	6	IVFMFLVTSA						
	117	FADKPDESTL						
	27	ASATGGASKL						
	100	RLDQEFNRKM						
	35	KLSCVTVWGL						
HLA-DR1			108	KMQEQNAKF				
			139	RMIKEHTEK				
			159	FKQKFAELL				
			170	QKAAQYPSK				
			147	KFNKKMHEH				
			150	KKMHEHSEH				
			129	EMREHYEKF				
			136	KFERMIKEH				

The results contain residues numbers and peptide sequence, which have been analyzed online by using four different HLA epitopes prediction systems. The results that are simultaneously predicted by three systems are marked with underline.

**Table 5:** Prediction of H2-D epitopes of Amastin-Kmp-11, Amastin-Gp63 and Kmp-11-Gp63.

Analysis tools	Amastin-Kmp-11		Amastin-Gp63		Kmp-11-Gp63	
Rankpep	4	SCIVFMFLV	287	AGYYSALTM	308	AGYYSALTM
	1	MLCSCIVFM	139	DGLSNTDFV	160	DGLSNTDFV
			4	SCIVFMFLV	197	VGVINIPAA
			1	MLCSCIVFM		
			176	VGVINIPAA		
SYFPEITHI	109	MQEQNAKFF	139	DGLSNTDFV	160	DGLSNTDFV
	86	SMATTYEEF	287	AGYYSALTM	197	VGVINIPAA
	159	FKQKFAELL	176	VGVINIPAA	308	AGYYSALTM
	28	SATGGASKL	4	SCIVFMFLV	73	FKQKFAELL
	151	KMHEHSEHF	152	SVPSEGDVL	173	SVPSEGDVL
	42	WGLKNDCNA	263	GAGSAGSHI	284	GAGSAGSHI
	132	EHYEKFERM	28	SATGGASKL	269	YGCGTLEYL
	172	AAQYPSKKL	232	VPVINSSTA	65	KMHEHSEHF
			272	KMRNAQDEL	253	VPVINSSTA
			42	WGLKNDCNA	293	KMRNAQDEL
			84	GGSEKRDIL	46	EHYEKFERM
			100	ALQLHTERL	23	MQEQNAKFF

The results contain residues numbers and peptide sequence, which have been analyzed online by using two different HLA epitopes prediction systems. The results that are simultaneously predicted by two systems are marked with underline.

### Preparation of DNA and protein vaccines

**Fig 2:**
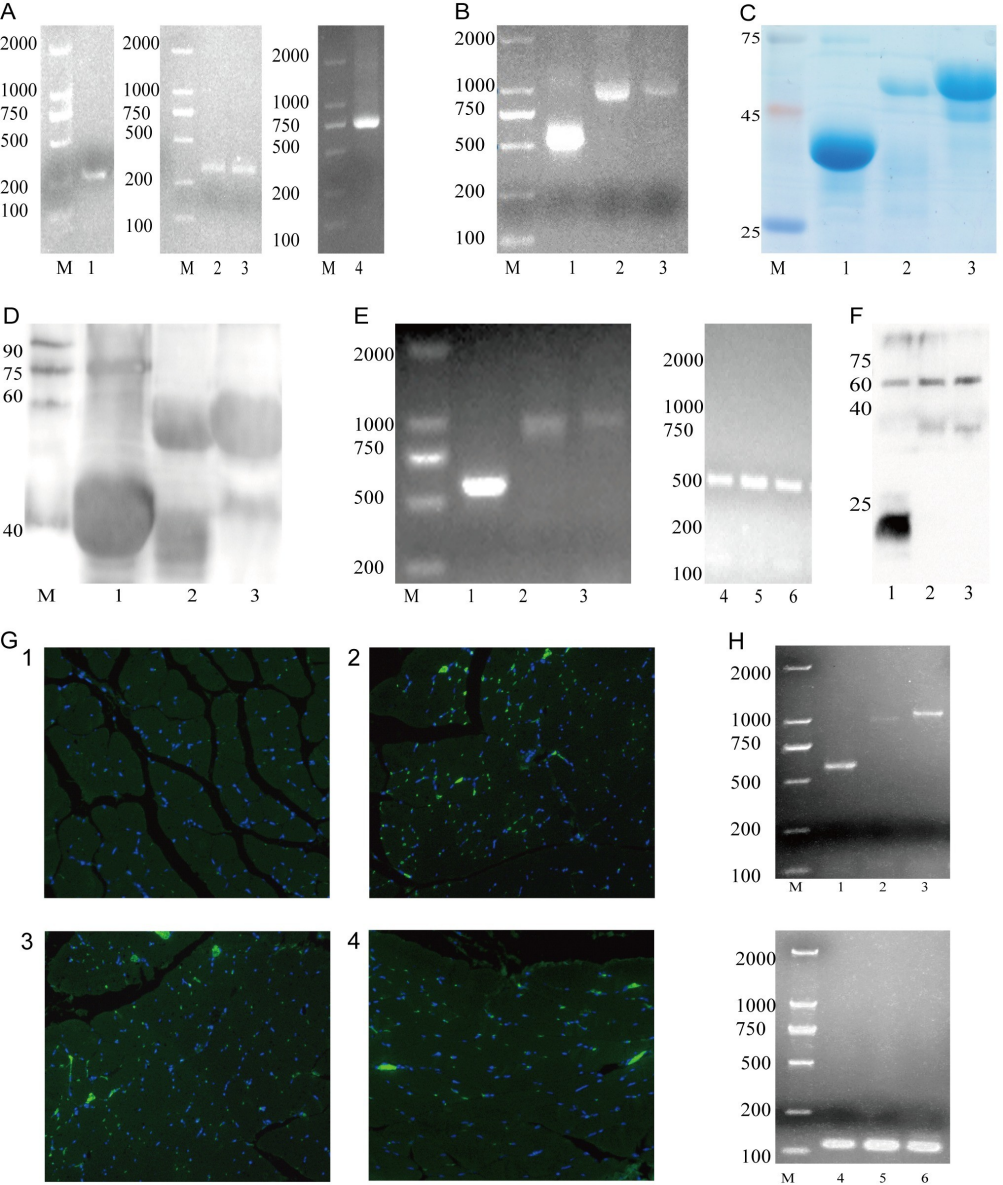
Detecting the target gene and its expression in prokaryotes and eukaryotes. (A) Prior genes were amplified by PCR. Lane M: DNA marker (DL2000), lane 1: a-linker (258bp), lane 2: k-linker (322bp) and lane 3: linker-k (322bp) and lane 4: linker-g (714bp). (B) B: Double combined genes were amplified by overlap PCR. Lane M: DNA marker (DL2000), lane 1: a-k (504bp), lane 2: a-g (897bp) and lane 3: k-g (963bp). (C, D) SDS-PAGE and Western blot of prokaryotic expression. Lane M: protein marker, lane 1: purified multi-epitopes protein pAmastin-Kmp-11 (39.8KDa), lane 2: purified multi-epitopes protein pAmastin-Gp63 (52.13KDa) and lane 3: purified multi-epitopes protein pKmp-11-Gp63 (55.93KDa). (E) Double combined genes in NIH3T3 cells were amplified by Rt-PCR. Lane M: DNA marker (DL2000), lane 1: a-k (585bp), lane 2: a-g (978bp), lane 3: k-g (1041bp) and lane 4–6: Rt-PCR amplification of GAPDH from three groups above, respectively. (F) Western blot of eukaryotic recombinant plasmid expression in NIH3T3. Lane 1: pVAX1-a-k (20.8KDa), lane 2: pVAX1-a-g (36.13KDa) and lane 3: pVAX1-k-g (37.2KDa). (G) Immunofluorescence of eukaryotic recombinant plasmids expression in BALB/c mice hind leg quadriceps. (1): G2, PBS injection. (2): G3, pVAX1-a-k injection. (3): G4, pVAX1-a-g injection. (4): G5, pVAX1-k-g injection. (H) Double combined genes in BALB/c mice hind leg quadriceps were amplified by RT-PCR. M: DNA marker (DL2000), lane 1: a-k (585bp), lane 2: a-g (978bp), lane 3: k-g (1041bp) and lane 4–6: RT-PCR amplification of GAPDH from three groups above, respectively.

The epitope genes of a-linker (228bp), k-linker (292bp), linker-k (292bp) and linker-g (684bp) were successfully obtained by PCR amplification (Fig 2A). The linked genes of a-k (559bp), a-g (927bp) and k-g (945bp) were amplified by overlap PCR (Fig 2B). SDS-PAGE and Western blot were performed to identify prokaryotic expression of the purified multi-epitopes protein (pAmastin-Kmp-11 39.8KDa, pAmastin-Gp63 52.13KDa and pKmp-11-Gp63 55.93KDa, with Trx-tag fusion [46–47]) (Fig 2C and 2D), of which molecular weights were in accordance with the results predicted by DNAstar software. The results of RT-PCR (Fig 2E) showed that the target genes a-k, a-g and k-g were successfully transcribed into RNA in cells. The results of Western blot (Fig 2F) demonstrated successful eukaryotic expression of the three target proteins pVAX1-a-k (20.8KDa), pVAX1-a-g (36.1KDa) and pVAX1-k-g (37.2KDa) in cells. Immunofluorescence (Fig 2G:1–4) showed that the result of PBS (G2) infection was negative, but the pVAX1-a-k (G3), pVAX1-a-g (G4) and pVAX1-k-g (G5) injection show positive. Double combined genes (a-k, a-g and k-g) were successfully detected by RT-PCR (Fig 2H). It was successfully proved by Immunofluorescence and RT-PCR that eukaryotic recombinant plasmids could express in BALB/c mice hind leg quadriceps.

### Specific lgG, lgG1, lgG2a antibody titers and cytokines levels

**Fig 3:**
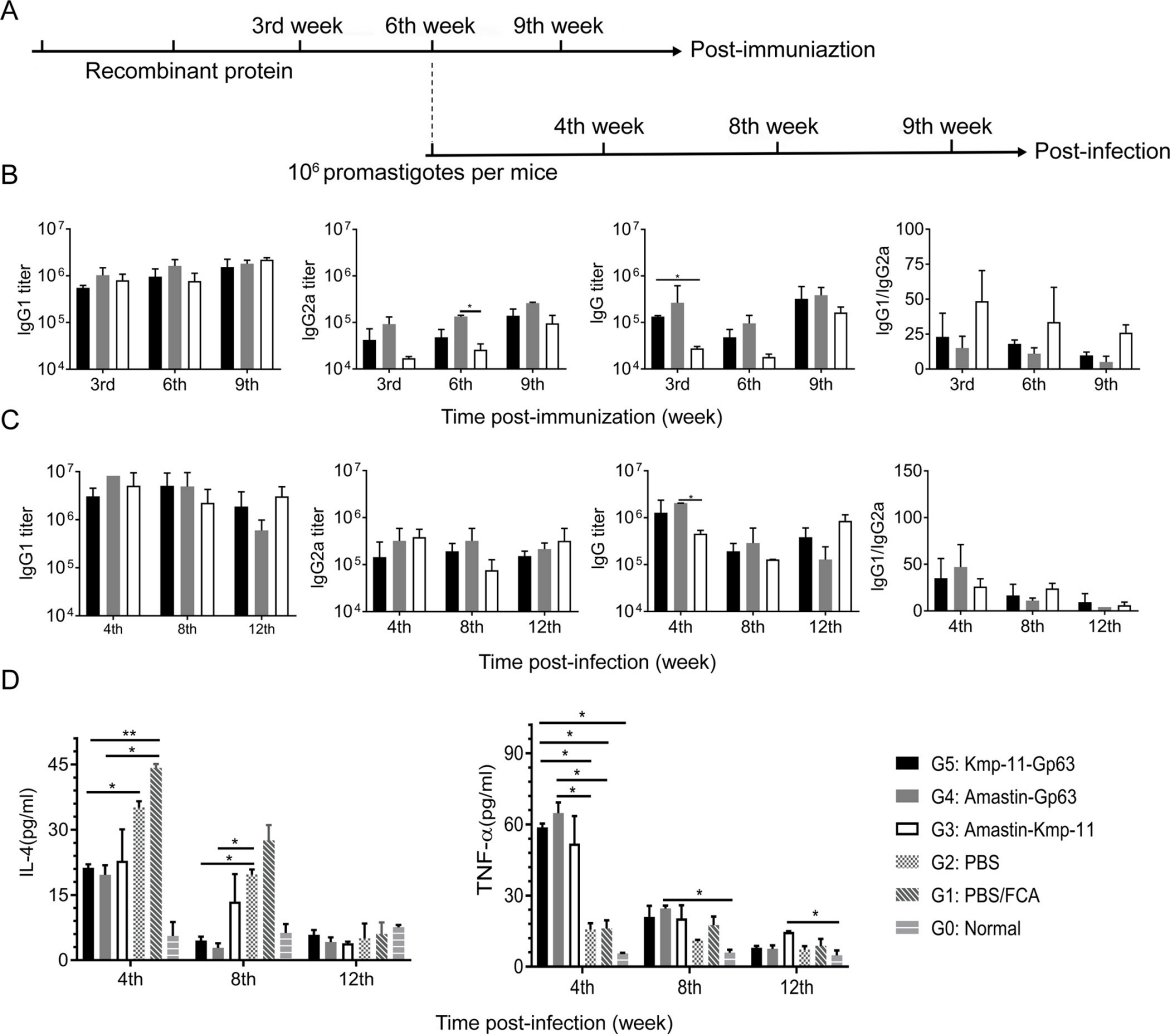
Immunization and challenge schedule, IgG titers and cytokine levels. (A) Three weeks after DNA intramuscular injection, protein boosted immunization was carried out. Mice were sacrificed for immune analysis at 3rd, 6th and 9th week post-immunization. In addition, infections were performed, and mice were euthanized for immune analysis at 4th 8th and 12th week post-infection. (B, C) Post-immunization or post-infection IgG, IgG1 and IgG2a antibody titers were measured by ELISA, and the values of IgG1/IgG2a were calculated as well. (D) Post-infection cytokines, IL-4 and TNF-α, were determined by ELISA. The data were presented as mean ± SD. Statistical analyses were performed by IBM SPSS Statistics 22 with one-way analysis of variance, and the significant difference was designed as asterisks (*P<0.05, **P<0.01,***P<0.001).

ELISA was performed to evaluate specific lgG, lgG1, lgG2a titers and cytokines IL-4 and TNF-α levels in serum. As shown in Fig 3B, IgG1 titers of the three vaccine groups were >10^5^ and gradually increased at 3rd, 6th and 9th week post-immunization. IgG2a titers of the three vaccine groups were >10^4^ and also increased with time. The total IgG titers of Amastin-Gp63 group were >10^5^ at 3rd, 6th and 9th week post-immunization. However, the total IgG titers of Amastin-Kmp-11 group only exceeded 10^5^ at the 9th week post-immunization. The ratios of IgG1 to IgG2a showed that the immune responses induced by the three vaccines were more inclined to humoral immunity.

As shown in Fig 3C, IgG1 titers of the three groups were more than 10^6^ at 4th and 8th week post-infection and gradually decreased over time. IgG2a titers of Amastin-Gp63 and Kmp-11-Gp63 groups kept stable levels and exceeded 10^5^. The total IgG titers of the three groups were also more than 10^5^ at 4th, 8th and 12th week post-infection. Compared with the results after immunization, the titers of total IgG, IgG1 and IgG2a increased in each vaccine group after infection. The ratios of IgG1 to IgG2a post-infection in each group gradually decreased over time.

Compared with the results of normal, PBS/FCA and PBS groups, the IL-4 and TNF-α levels of the three vaccine groups were significantly different (Fig 3D). The IL-4 levels of the three vaccine groups were significantly lower than those of control groups at 4th and 8th week post-infection, and the TNF-α levels of the three vaccine groups were significantly higher than those of normal and control groups at 4th and 8th week post-infection, which meant the efficacy of our vaccines. Among the three epitope-based vaccine groups, the IL-4 levels of Amastin-Gp63 group were the lowest, and the TNF-α levels of that were the highest at 4th and 8th week post-infection.

### Detection of CD4^+^ and CD8^+^ lymphocytes in the spleen by flow cytometry

To learn about CD4^+^ and CD8^+^ lymphocytes in spleen, splenocytes were analyzed using flow cytometry. As shown in S1A and S1B Fig, after infection, the percentage of CD3^+^CD4^+^ and CD3^+^CD8^+^ lymphocyte from normal, PBS/FCA and PBS groups was 11.24% and 8.12%, 14.81% and 9.67%, 14.75% and 9.94%, respectively. The percentage of CD3^+^CD4^+^ and CD3^+^CD8^+^ lymphocyte in Amastin-Kmp-11 and Kmp-11-Gp63 groups was 16.46% and 10.24%, 15.96% and 9.04%, respectively. Amastin-Gp63 group had the largest percentage of CD3^+^CD4^+^ and CD3^+^CD8^+^ lymphocyte, accounting for 18.3% and 13.06%. Double negative T cells (DN T cells) due to their CD3^+^CD4^-^CD8^-^ were evaluated according to the gates of B-+, C-+, B++ and C++ in S1C Fig at 8th week post-infection. The percentages of DN T cells in vaccinated groups were higher than control groups, and Kmp-11-Gp63 and Amastin-Gp63 groups were significantly higher than PBS group.

### Hepatic histopathology analysis

**Fig 4:**
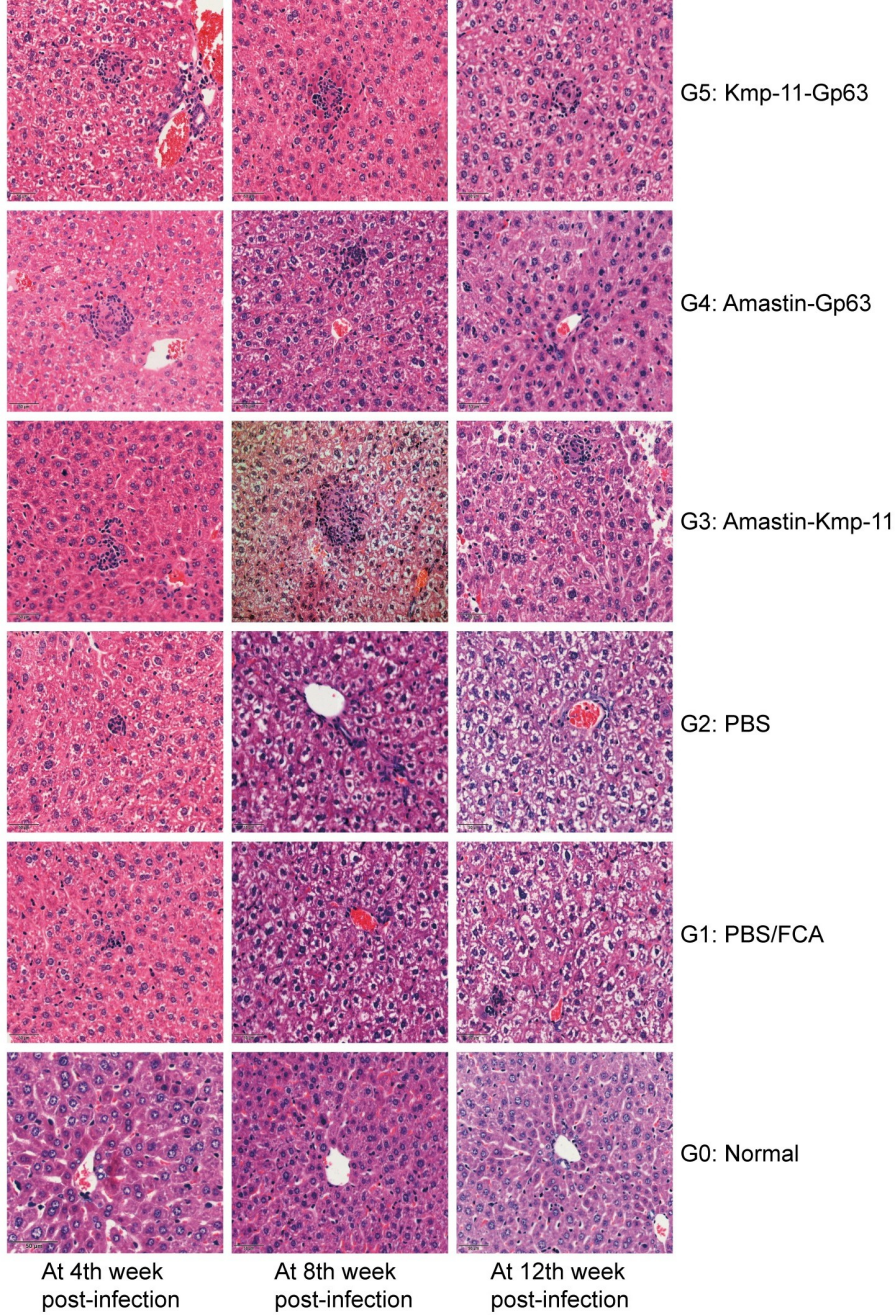
Histopathology of livers analysis. Livers tissues from groups at 4th, 8th and 12th week post-infection were obtained, fixed and stained with haematoxylin-eosin. The sections were observed by using an optical microscope at 400 times the original magnification.

As shown in Fig 4, at 4th week post-infection, Amastin-Gp63 and Kmp-11-Gp63 groups had formed mature granulomas, and two control groups (PBS/FCA and PBS group) and Amastin-Kmp-11 groups had the only infiltration of inflammatory cells. At 8th week post-infection, there were mature granulomas in Amastin-Kmp-11 group, and the granulomas of Amastin-Gp63 group begun to dissipate. Moreover, different degrees of hepatocyte swelling occurred in all infected groups, and the most severe swelling was in the PBS group. At 12th week post-infection, the three vaccine groups showed a lesser degree of cell swelling compared to PBS/FCA and PBS groups, and Amastin-Gp63 group showed regression of granulomas. As for PBS/FCA and PBS groups, granuloma was still not found, and the cellular swelling was the most severe among all groups. Immature and mature granulomas were counted in 100 fields under 400 power magnification. As shown in S2 Fig, in the count of immature and mature granulomas, the number of granulomas in each group increased with infection time. At almost every time point, the number of granulomas in the vaccinated groups was larger than that in the control group. At 8th week post-infection, the average number of immature granuloma in Amastin-Gp63 was significantly higher than that in PBS group, and the average number of mature granuloma in Amastin-Gp63 was significantly higher than that in Amastin-kmp-11 group at 4th week post-infection.

### Spleen smears analysis and parasite burdens

**Fig 5:**
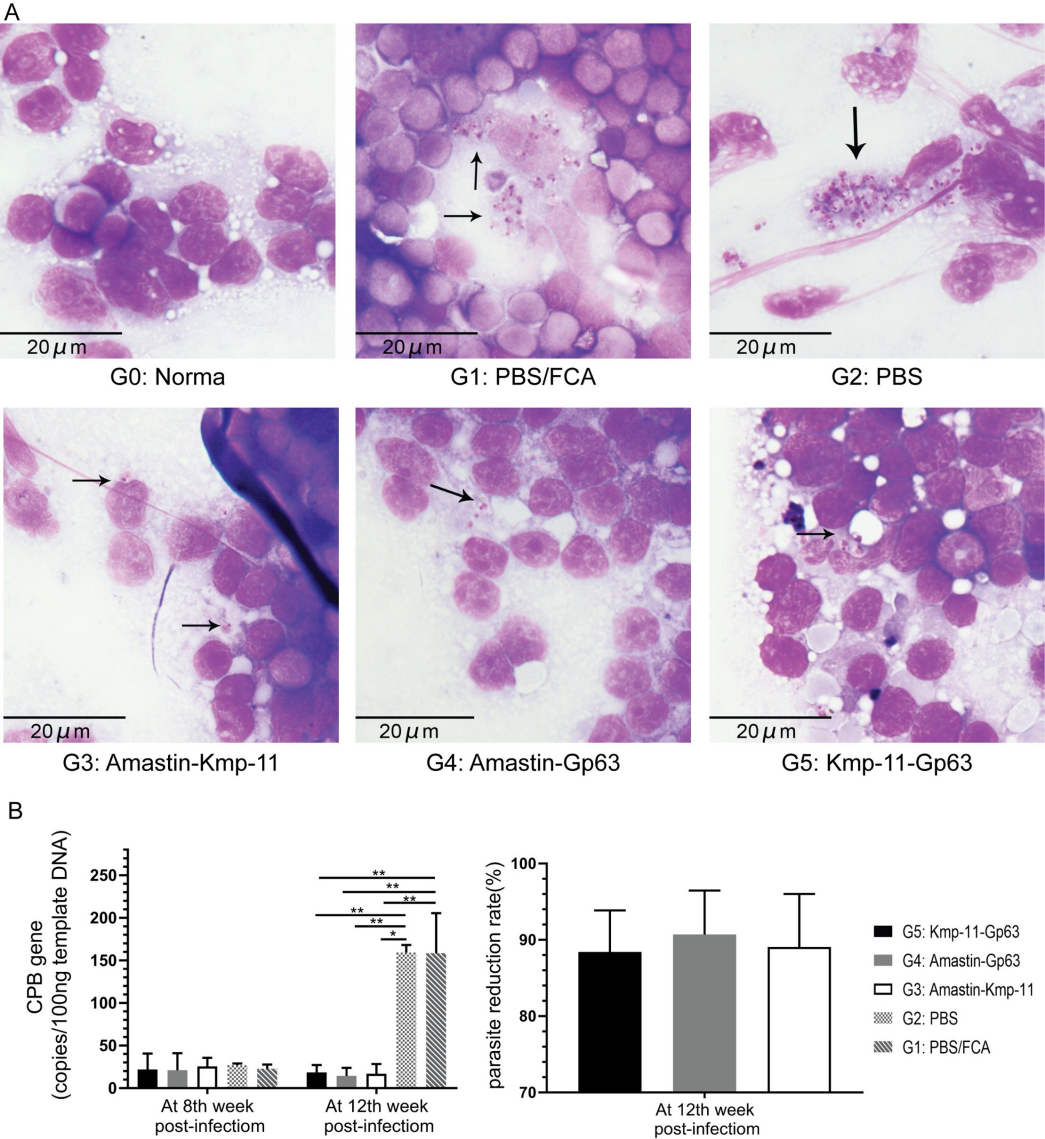
Parasite burdens were determined by smears and DNA qPCR. (A) Spleens from each group were collected for Swiss-stained smears and parasites were marked with arrows. (B) DNA from each group of spleens was extracted for amplification of CPB gene by qPCR to identify the parasite burdens. The values of parasite reduction rate were calculated as well. The data were presented as mean ± SEM. Statistical analyses were performed by IBM SPSS Statistics 22 with one-way analysis of variance, and the significant difference was designed as asterisks (*P<0.05, **P<0.01,***P<0.001).

To confirm the infection, at 12th week post-infection, spleen smears of all groups were made, and the findings were shown in Fig 5A. The amastigotes of *Leishmania* parasites were marked with arrows in the figure. A large number of parasites were found in PBS and PBS/FCA groups, while fewer parasites were found in the three vaccine groups.

As shown in Fig 5B, qPCR was performed to evaluate parasite burdens in the spleen. At the 8th week after infection, the CPB gene copies in 100 ng of template DNA of the three vaccine groups were approximately the same as the CPB gene copies of control groups. At 12th week post-infection, the average copy numbers of CPB in Amastin-Kmp-11, Amastin-Gp63 and Kmp-11-Gp63 groups were respectively 18.28, 14.78 and 16.65 copies/per 100 ng template DNA. These CPB copies of the three vaccine groups were significantly less than those of PBS/FCA and PBS groups (158.77 and 159.01 copies/per 100 ng template DNA, respectively). At 12th week post-infection, we also calculated the parasite reduction rates of the three vaccines and found that the average parasite reduction rates of Kmp-11-Gp63, Amastin-Gp63 and Amastin-kmp-11 groups were 88.42%, 91.01% and 89.38%, respectively.

## Discussion

In this study, we used a DNA prime-protein boost immunization strategy to explore the efficacy of dominant multi-epitopes DNA and protein vaccines for visceral leishmaniasis. We selected a new isolated clinical strain of *Leishmania infantum* with strong virulence for infection experiments as other strains kept in vitro for long-term may lost their virulence [48]. Amastin, Kmp-11 and Gp63 protein from the new strain were analyzed by bioinformatics tools to explore their secondary structural characteristics, HLA and H2 restricted epitopes, ligand binding sites and tertiary structures. On the basis of silico analytic results, dominant epitopes of the three proteins were selected and analyzed to reduce the adverse effects of unrelated peptide [49]. Studies have shown that the immune effect of multiple antigen combinations is often stronger than a single one [50]. Therefore, we reorganized the selected antigens in pairs and successfully employed the optimal epitopes to construct dominant multi-epitopes DNA and protein vaccines against VL that performed good immunoprotection.

The secondary structures and surface properties of proteins affect their immunogenicity, such as α-helix, β-sheet, β-turn, coil region, and hydrophilicity and flexibility of peptide [49, 51, 52]. Based on the results of bioinformatics analysis of Amastin, Kmp-11 and Gp63 proteins, the epitope-based vaccine Amastin-Gp63, Amastin-Kmp-11 and Kmp-11-Gp63 had a high antigenic index and they were rich in HLA-A24, HLA-A2, HLA-DR1 and H2-D epitopes. The existence of potential binding sites in Amastin-Gp63 and Kmp-11-Gp63 works on the interaction of epitopes with the antigen-presenting cells [53, 54], suggesting its immunogenicity. HLA class Ⅰ molecules are involved in the presentation of endogenous antigens, activate CD8^+^ cells, and participate in cellular immunity. HLA class Ⅱ molecules present exogenous antigens to activate CD4^+^ cells and regulate immune responses. Among the three epitope-based vaccines, Amastin-Gp63 contained the largest number of HLA-A2, HLA-A24, HLA-DR1 and H2-d epitopes, and its immunogenicity was originally thought to be better than that of Amastin-Kmp-11 and Kmp-11-Gp63. Compared with the results of Alonso-Padilla et al [52, 55], the three vaccines in our study had a relatively large number of HLA-A2, HLA-A24, HLA-DR1 and H2-d epitopes, suggesting that these epitopes might serve as the basis for supporting humoral and cellular immunity in animal experiments.

In animal experiments, after immunization, lgG1 titers were consistently higher than 10^5^, and the titers of IgG and IgG2a at 3rd and 9th week were also higher than 10^5^. After infection, all kinds of antibody titers had maintained a high level for long-term because of the effect of the immunization. The results of IgG1 and IgG2a titers suggested that our vaccines cause high levels of Th2 and Th1 responses post-infection, and Th2 responses were dominant. By comparing the levels of IL-4 and TNF-α in the control and vaccinated groups post-infection, it was found that the vaccines might regulate the Th1 and Th2 responses to perform the leishmanicidal actions.

CD4^+^ and CD8^+^ T cells play vital protective roles in fighting leishmaniasis and protecting the host from damage. CD8^+^ T cells are capable of recognizing intracellular pathogens through identifying internal proteins of pathogens that are presented on the surface of antigen-presenting cells [56]. CD8^+^ T cells can produce Th1 cytokines to activate macrophage killing *Leishmania* parasites [57]. CD4^+^ T cells can differentiate into IFN-γ-secreting Th1 cells and IL-17-secreting Th17 cells that can induce and maintain CD8 responses [56], and activate macrophage to kill amastigotes. Meanwhile, CD4^+^ T cells are induced to be IL-4 and IL-10-secreting Th2 cells and Treg cells. Treg cells produce suppressive cytokines IL-10 and TGF-β, which control excessive detrimental inflammatory responses [10]. At the chronic infection in spleen, the apoptotic deletion caused by progressive and sustained expression of inhibitory receptors on effector T cells, leads to T cell exhaustion, especially CD8^+^ T cells [10, 58]. CD4^+^ and CD8^+^ T cells also produce TNF-α that plays a crucial role in the maturation of granulomas in livers. Double negative T cells (CD3^+^CD4^-^CD8^-^, DN T cells), a small subpopulation of CD3^+^ T lymphocytes, appear in secondary lymphoid organs and peripheral blood of healthy individuals including human and mice, which contributes to physiological immunological responses against intracellular pathogens, influence long-term allograft survival and prevent autoimmune disease [59–61]. As for the performance of DN T cells in immune response against *Leishmania*, it’s reported that DN T cells display functional characteristics of anti-*Leishmania* memory-like cells that are highly proliferative and can produce IFN-γ and TNF to mediate immune response [61]. DN T cells play an important role in primary and vaccine-induced immunity against *Leishmania* [61]. According to the results of flow cytometry from spleen, mice in vaccine groups showed higher percentages of CD4^+^ and CD8^+^ T cells than those in the normal and control groups at 8th week post-infection. Unfortunately, there was no significant difference in the percentages of CD4^+^ and CD8^+^ T cell between control and vaccinated groups, possibly due to the low dose and frequency of immunization. In our study result of DN T cells, we also found that the percentages of DN T cell in vaccinated groups were more than control group, which were consistent with the results of CD4^+^ and CD8^+^ T cell and indicated the effectiveness of our vaccines.

After *Leishmania* parasites infection in mice, the immune responses of liver and spleen are different, presenting a compartmentalized immune reaction [10]. The liver usually relies on immune responses and granulation formation to clear parasites. In contrast, the immune system of spleen fails to clear parasites and instead, a long-term chronic infection persists [62–64]. Therefore, we made liver tissue sections and spleen smears and detected spleen parasite burdens to evaluate the immunoprotection of the vaccines. Hepatic granuloma, composed of Kupfer cells, Langhans cells, neutrophils and various types of chemotactic T cells, plays an essential role in parasites clearance and is considered as a hallmark of immunoprotection [65, 66]. Mature granulomas usually form at 4th week post-infection [10]. The vaccine groups except Amastin-Kmp-11 group showed mature hepatic granulomas with a little cellular swelling of hepatocytes in the early stage of the disease and mild swelling of hepatocytes in the later stage. The control groups lacked mature granulomas in the liver with some spotted necrosis at 4th week post-infection and severe cellular hepatocyte swelling at 8th and 12th week post-infection. Mature hepatic granulomas appeared in Amastin-Gp63 group were consistent with the early high secretion of TNF-α. The pathological changes of liver were consistent with the results in earlier studies [65, 67, 68], suggesting the immunoprotection of our vaccines. In addition, TNF-α is also of great importance to leucocyte recruitment, which makes contributions to granulomas formation [9]. Our results also demonstrated the fact that the number of liver immature granulomas was basically consistent with the trend of TNF-α level at 4th, 8th and 12th week post-infection.

Based on the results of spleen smear observation and spleen parasite burden detection, the three vaccines showed good immunoprotection, and the Amastin-Gp63 epitope-based vaccine was the best. Because of the main histocompatibility complex *Lsh* mutation in BALB/c mice, the natural resistance to *Leishmania* parasites is influenced so that the proliferation of the parasites is unrestrained, which leads to the susceptibility of BALB/c mice [62, 69]. In this study, each group of mice developed into chronic infection with increasing parasite burdens in spleen over time, especially in the control groups, which were consistent with other studies and suggested that these vaccines could slow down the progression of leishmaniasis in BALB/c mice model [70, 71].

In summary, we successfully constructed three dominant multi-epitopes DNA and protein vaccines, which were demonstrated to have good immunogenicity and immunoprotection, and Amastin-Gp63 vaccine worked best. This study may provide a reference for other *Leishmania* vaccine studies, laying a foundation for future studies on dominant DNA/protein and epitope-based vaccines.

## Supporting information

S1 Fig Detection of CD4^+^ and CD8^+^ lymphocytes in the spleen by flow cytometry. As shown in S1B Fig, the levels of CD3^+^CD4^+^ and CD3^+^ CD8^+^ lymphocyte in spleen from all groups were detected by flow cytometry at 3rd week post-immunization 8th week post-infection. In S1C Fig, the percentages of DN T cells from vaccinated groups and control groups were evaluated.
(TIF)

S2 Fig Quantification of immature and mature granuloma. The hepatic number of immature and mature granuloma in 100 fields at 400 magnification were quantified at 3rd, 8th and 12th post-infection.
(TIF)

S1 Table The amino acid sequences of different systems expression. (DOCX)

S1 Raw images The original images for blots and gels. (PDF)
