# Supplementary figures and images for: The immunogenicity and protective immunity of multi-epitopes DNA prime-protein boost vaccines encoding Amastin-Kmp-11, Kmp11-Gp63 and Amastin-Gp63 against visceral leishmaniasis
Source: PLoS One. 2020 Mar 16;15(3):e0230381. doi: 10.1371/journal.pone.0230381 (PMC7075555; doi:10.1371/journal.pone.0230381)

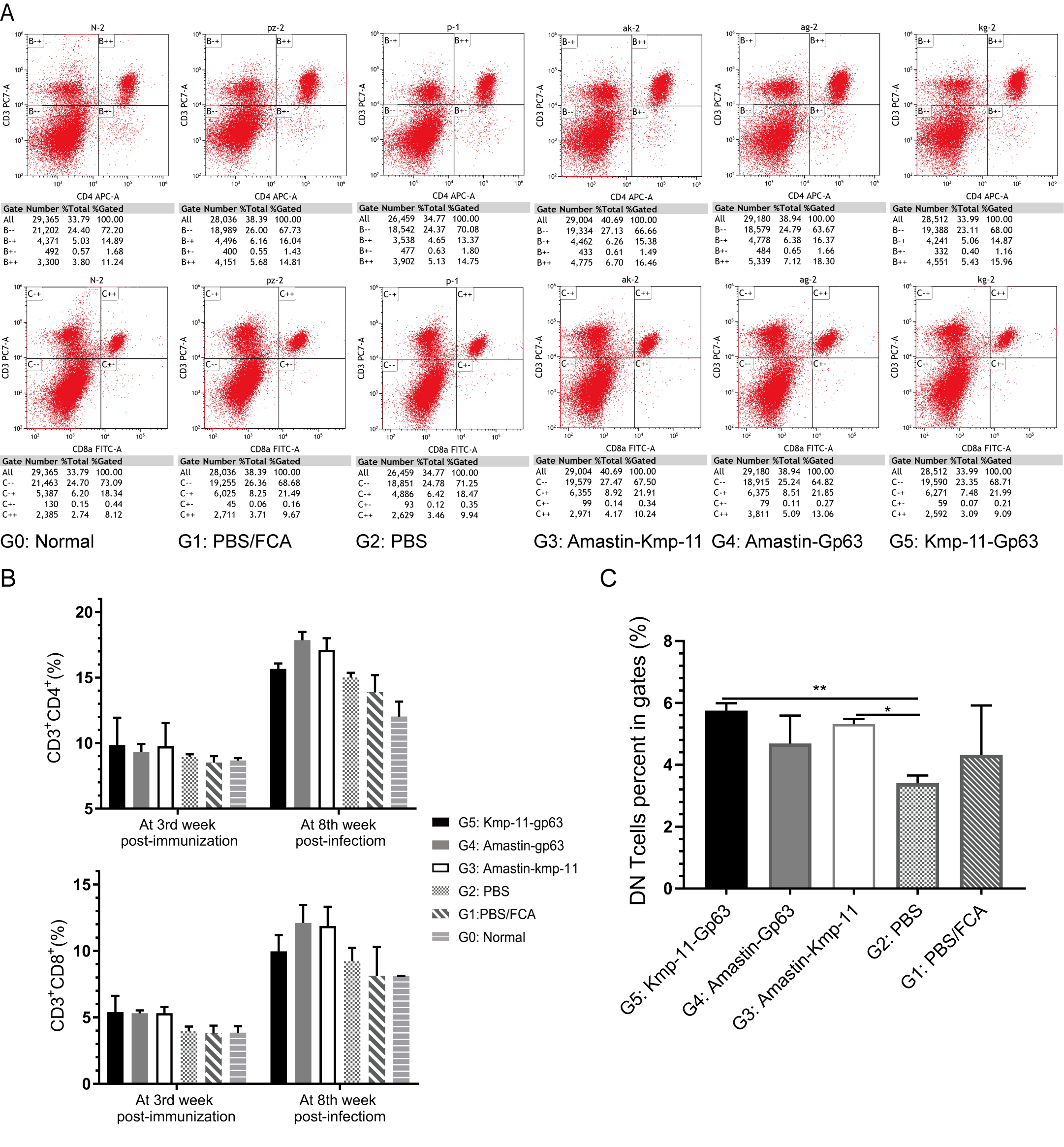

Supplement: S1 Fig — As shown in S1B Fig, the levels of CD3+CD4+ and CD3+ CD8+ lymphocyte in spleen from all groups were detected by flow cytometry at 3rd week post-immunization 8th week post-infection. In S1C Fig, the percentages of DN T cells from vaccinated groups and control groups were evaluated. (TIF) [file pone.0230381.s001.tif]

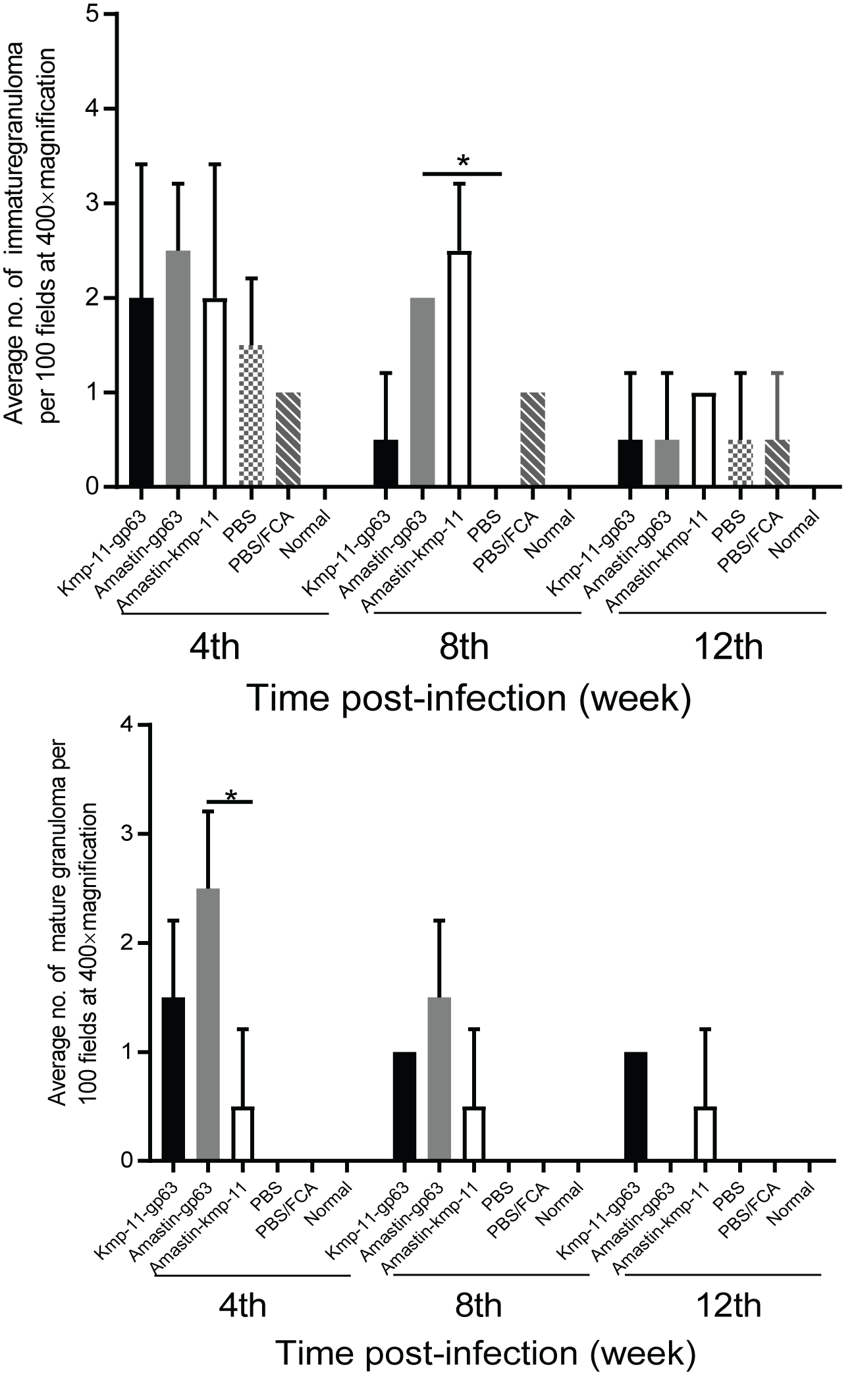

Supplement: S2 Fig — The hepatic number of immature and mature granuloma in 100 fields at 400 magnification were quantified at 3rd, 8th and 12th post-infection. (TIF) [file pone.0230381.s002.tif]
