# Supplementary material for: The immunogenicity and protective immunity of multi-epitopes DNA prime-protein boost vaccines encoding Amastin-Kmp-11, Kmp11-Gp63 and Amastin-Gp63 against visceral leishmaniasis
Source: PLoS One. 2020 Mar 16;15(3):e0230381. doi: 10.1371/journal.pone.0230381 (PMC7075555; doi:10.1371/journal.pone.0230381)
Supplement: S1 Table — (DOCX) [file pone.0230381.s003.docx]

**S2 Table. The amino acid sequences of different systems expression.**

| Different systems expression | Double combined gene | Molecular Weight | Amino acid sequence |
| --- | --- | --- | --- |
| The initial amino acid sequences of bacterial system expression | Amastin-Kmp-11 | 39  KDA | MSDKIIHLTDDSFDTDVLKADGAILVDFWAEWCGPCKMIAPILDEIADEYQGKLTVAKLNIDQNPGTAPKYGIRGIPTLLLFKNGEVAATKVGALSKGQLKEFLDANLAGSGSGHMHHHHHHSSGLVPRGSGMKETAAAKFERQHMDSPDLGTDDDDKAMADIGS**MLCSCIVFMFLVTSAPISQFRGRGINASATGGASKLSCVTVWGLKNDCNANNYDYRPTSIGCARSKQLFQVGGGGSGGGGSGGGGSMATTYEEFSAKLDRLDQEFNRKMQEQNAKFFADKPDESTLSPEMREHYEKFERMIKEHTEKFNKKMHEHSEHFKQKFAELLEQQKAAQYPSK**KLAAALEHHHHHH. |
|  | Amastin-Gp63 | 52.3  KDA | MSDKIIHLTDDSFDTDVLKADGAILVDFWAEWCGPCKMIAPILDEIADEYQGKLTVAKLNIDQNPGTAPKYGIRGIPTLLLFKNGEVAATKVGALSKGQLKEFLDANLAGSGSGHMHHHHHHSSGLVPRGSGMKETAAAKFERQHMDSPDLGTDDDDKAMADIGS**MLCSCIVFMFLVTSAPISQFRGRGINASATGGASKLSCVTVWGLKNDCNANNYDYRPTSIGCARSKQLFQVGGGGSGGGGSGGGGSEKRDILVKYLIPQALQLHTERLKVRQVQDKWKVTGMGNEICGHFKVPPAHITDGLSNTDFVMYVASVPSEGDVLAWATTCQVFSDGHPAVGVINIPAANIASRYDQLVTRVVTHEMAHALGFSVVFFRDARILESISNVRHKDFDVPVINSSTAVAKAREQYGCGTLEYLEMEDQGGAGSAGSHIKMRNAQDELMAPASDAGYYSALTMAIFQDLGFYQADFS**KLAAALEHHHHHH. |
|  | Kmp-11-Gp63 | 55.9  KDA | MSDKIIHLTDDSFDTDVLKADGAILVDFWAEWCGPCKMIAPILDEIADEYQGKLTVAKLNIDQNPGTAPKYGIRGIPTLLLFKNGEVAATKVGALSKGQLKEFLDANLAGSGSGHMHHHHHHSSGLVPRGSGMKETAAAKFERQHMDSPDLGTDDDDKAMADIGS**MATTYEEFSAKLDRLDQEFNRKMQEQNAKFFADKPDESTLSPEMREHYEKFERMIKEHTEKFNKKMHEHSEHFKQKFAELLEQQKAAQYPSKGGGGSGGGGSGGGGSEKRDILVKYLIPQALQLHTERLKVRQVQDKWKVTGMGNEICGHFKVPPAHITDGLSNTDFVMYVASVPSEGDVLAWATTCQVFSDGHPAVGVINIPAANIASRYDQLVTRVVTHEMAHALGFSVVFFRDARILESISNVRHKDFDVPVINSSTAVAKAREQYGCGTLEYLEMEDQGGAGSAGSHIKMRNAQDELMAPASDAGYYSALTMAIFQDLGFYQADFS**KLAAALEHHHHHH. |
| The amino acid sequences of mammalian system expression | Amastin-Kmp-11 | 20.8  KDA | MG**MLCSCIVFMFLVTSAPISQFRGRGINASATGGASKLSCVTVWGLKNDCNANNYDYRPTSIGCARSKQLFQVGGGGSGGGGSGGGGSMATTYEEFSAKLDRLDQEFNRKMQEQNAKFFADKPDESTLSPEMREHYEKFERMIKEHTEKFNKKMHEHSEHFKQKFAELLEQQKAAQYPSKHHHHHH.** |
|  | Amastin-Gp63 | 34.1  KDA | MG**MLCSCIVFMFLVTSAPISQFRGRGINASATGGASKLSCVTVWGLKNDCNANNYDYRPTSIGCARSKQLFQVGGGGSGGGGSGGGGSEKRDILVKYLIPQALQLHTERLKVRQVQDKWKVTGMGNEICGHFKVPPAHITDGLSNTDFVMYVASVPSEGDVLAWATTCQVFSDGHPAVGVINIPAANIASRYDQLVTRVVTHEMAHALGFSVVFFRDARILESISNVRHKDFDVPVINSSTAVAKAREQYGCGTLEYLEMEDQGGAGSAGSHIKMRNAQDELMAPASDAGYYSALTMAIFQDLGFYQADFSHHHHHH.** |
|  | Kmp-11-Gp63 | 37.7  KDA | MG**MATTYEEFSAKLDRLDQEFNRKMQEQNAKFFADKPDESTLSPEMREHYEKFERMIKEHTEKFNKKMHEHSEHFKQKFAELLEQQKAAQYPSKGGGGSGGGGSGGGGSEKRDILVKYLIPQALQLHTERLKVRQVQDKWKVTGMGNEICGHFKVPPAHITDGLSNTDFVMYVASVPSEGDVLAWATTCQVFSDGHPAVGVINIPAANIASRYDQLVTRVVTHEMAHALGFSVVFFRDARILESISNVRHKDFDVPVINSSTAVAKAREQYGCGTLEYLEMEDQGGAGSAGSHIKMRNAQDELMAPASDAGYYSALTMAIFQDLGFYQADFSHHHHHH.** |

The bold type sequences are the sequences translated by double combined genes.
