# Supplementary material for: The immunogenicity and protective immunity of multi-epitopes DNA prime-protein boost vaccines encoding Amastin-Kmp-11, Kmp11-Gp63 and Amastin-Gp63 against visceral leishmaniasis
Source: PLoS One. 2020 Mar 16;15(3):e0230381. doi: 10.1371/journal.pone.0230381 (PMC7075555; doi:10.1371/journal.pone.0230381)
Supplement: S1 Raw images — (PDF) [file pone.0230381.s004.pdf]

1. The raw images of Fig 2A.

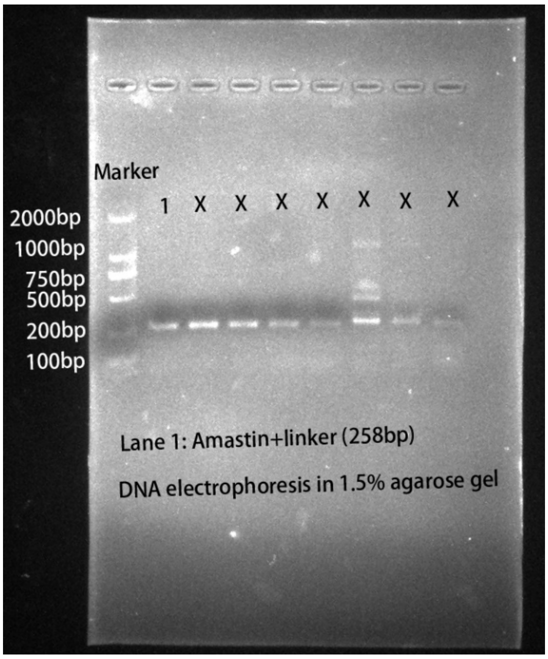

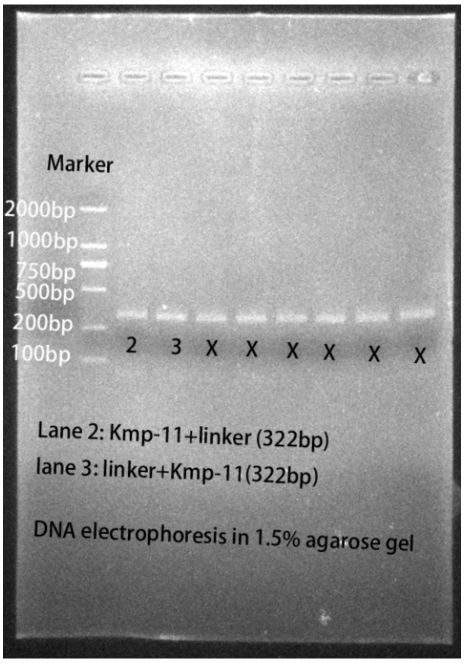

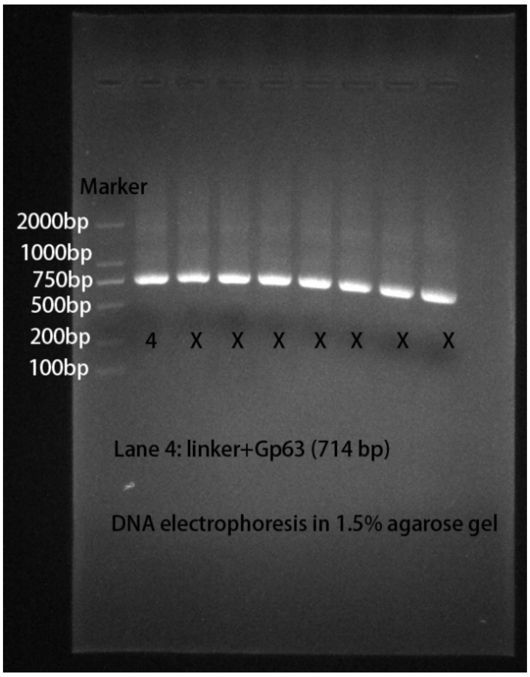

2. The raw images of Fig 2B.

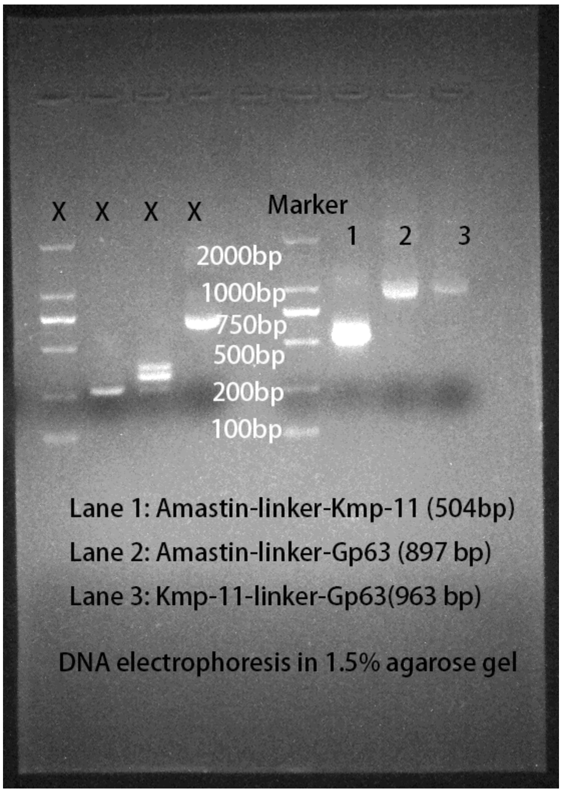

3. The raw images of Fig 2C.

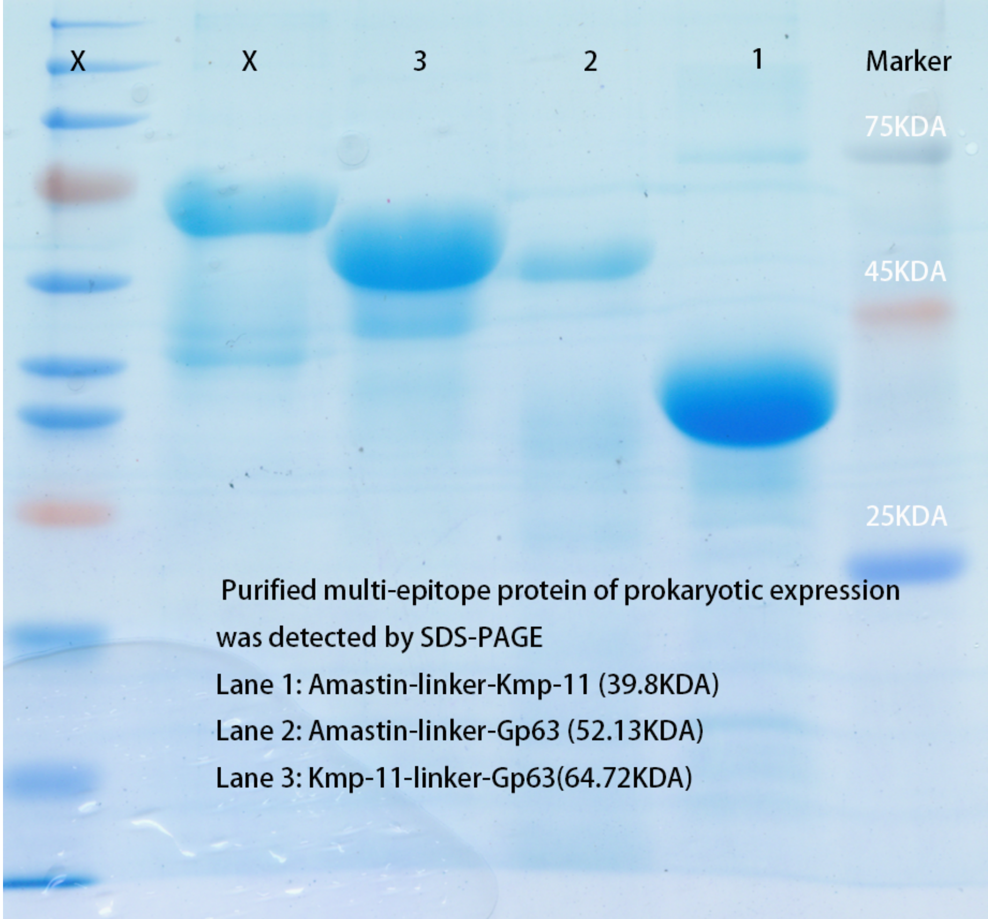

4. The raw images of Fig 2D.

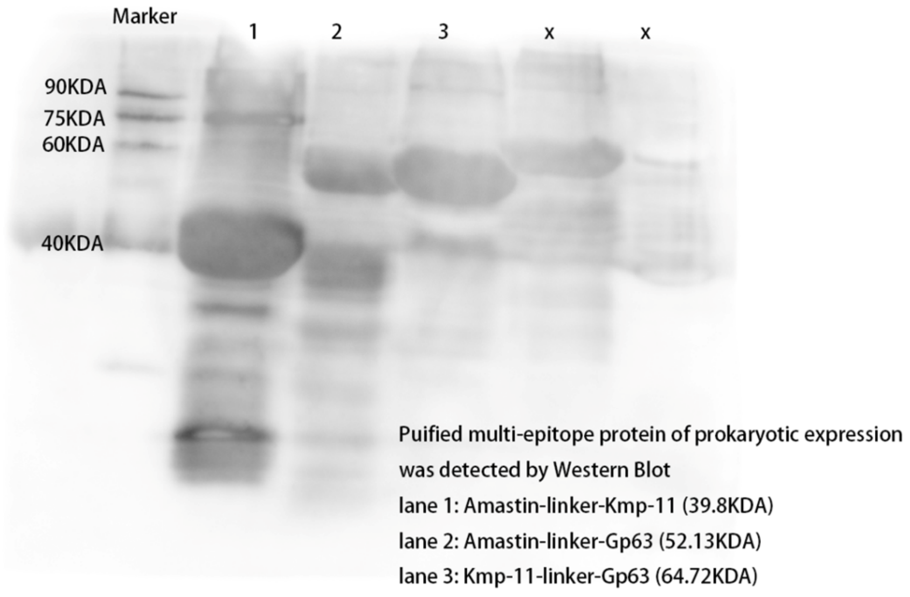

5. The raw images of Fig 2E.

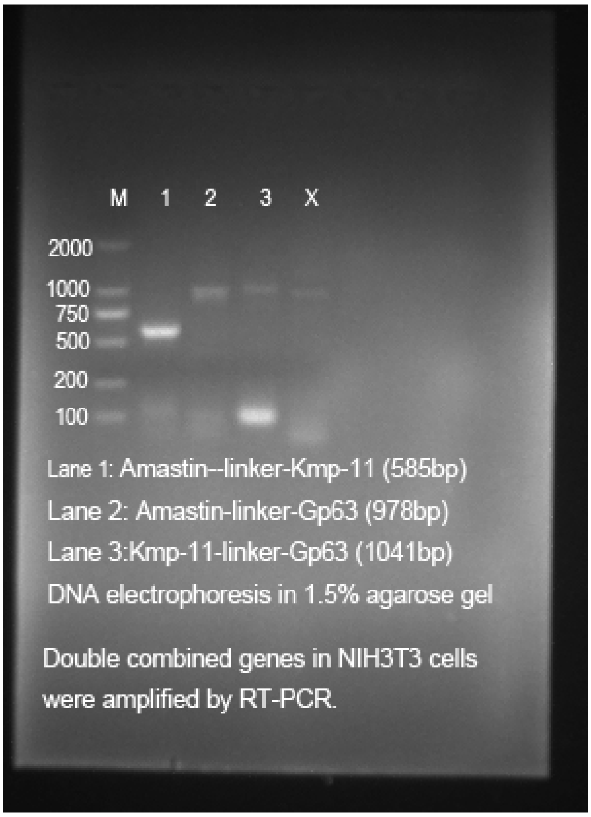

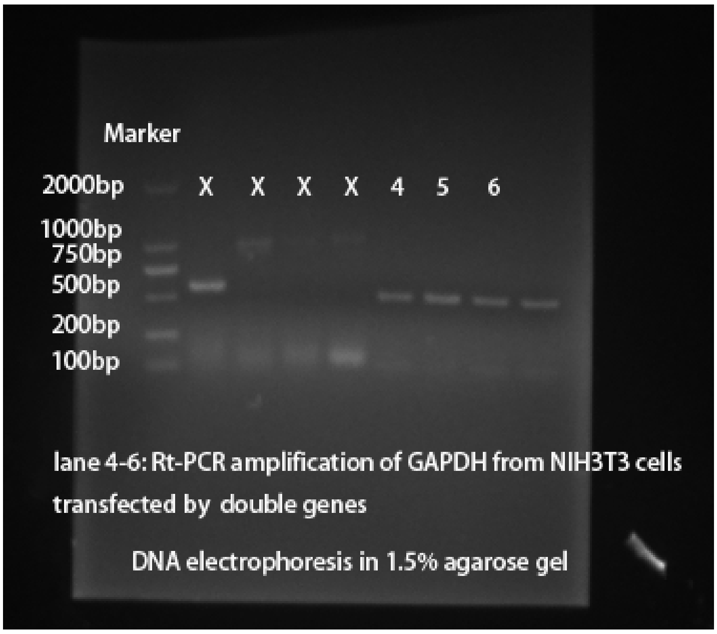

6. The raw images of Fig 2F.

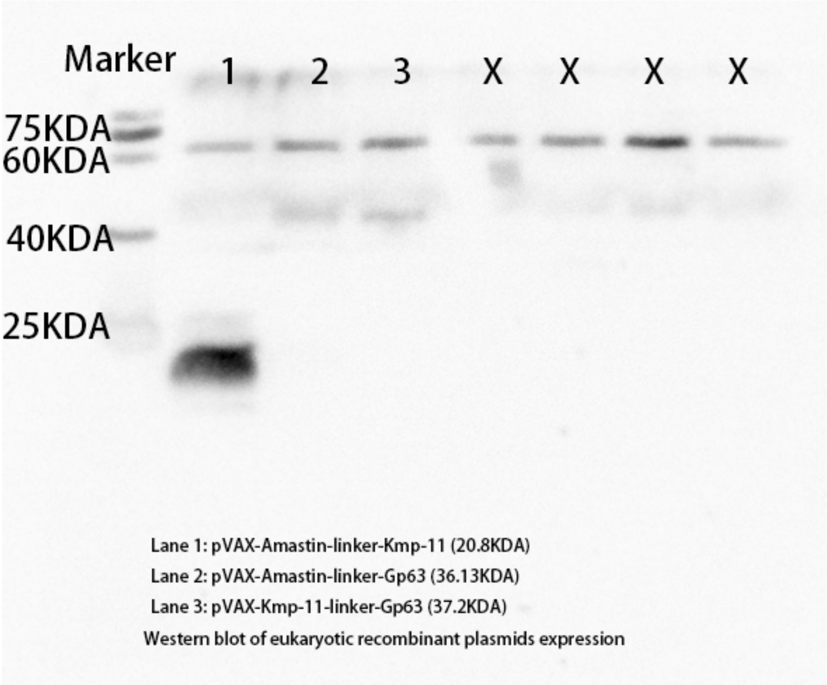

7. The raw images of Fig 2H.

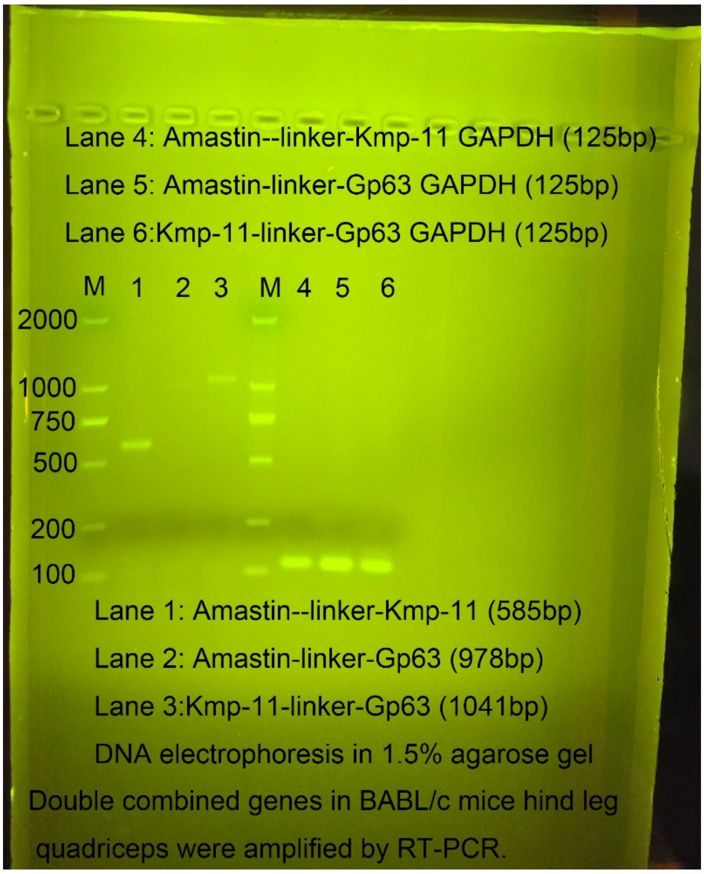
Lane 4: Amastin--linker-Kmp-11 GAPDH (125bp)
Lane 5: Amastin-linker-Gp63 GAPDH (125bp)
Lane 6:Kmp-11-linker-Gp63 GAPDH (125bp)
M 1 2 3 M 4 5 6
2000
1000
750
500
200
100
Lane 1: Amastin--linker-Kmp-11 (585bp)
Lane 2: Amastin-linker-Gp63 (978bp)
Lane 3:Kmp-11-linker-Gp63 (1041bp)
DNA electrophoresis in 1.5% agarose gel
Double combined genes in BABL/c mice hind leg
quadriceps were amplified by RT-PCR.
